# Amplicon-based semiconductor sequencing of human exomes: performance evaluation and optimization strategies

**DOI:** 10.1007/s00439-016-1656-8

**Published:** 2016-03-22

**Authors:** E. Damiati, G. Borsani, Edoardo Giacopuzzi

**Affiliations:** Unit of Genetics, Department of Molecular and Translational Medicine, University of Brescia, 25123 Brescia, Italy

**Keywords:** Variant Identification, Exome Sequencing, Whole Exome Sequencing, Torrent Variant Caller, High Confident Region

## Abstract

The Ion Proton platform allows to perform whole exome sequencing (WES) at low cost, providing rapid turnaround time and great flexibility. Products for WES on Ion Proton system include the AmpliSeq Exome kit and the recently introduced HiQ sequencing chemistry. Here, we used gold standard variants from GIAB consortium to assess the performances in variants identification, characterize the erroneous calls and develop a filtering strategy to reduce false positives. The AmpliSeq Exome kit captures a large fraction of bases (>94 %) in human CDS, ClinVar genes and ACMG genes, but with 2,041 (7 %), 449 (13 %) and 11 (19 %) genes not fully represented, respectively. Overall, 515 protein coding genes contain hard-to-sequence regions, including 90 genes from ClinVar. Performance in variants detection was maximum at mean coverage >120×, while at 90× and 70× we measured a loss of variants of 3.2 and 4.5 %, respectively. WES using HiQ chemistry showed ~71/97.5 % sensitivity, ~37/2 % FDR and ~0.66/0.98 F1 score for indels and SNPs, respectively. The proposed low, medium or high-stringency filters reduced the amount of false positives by 10.2, 21.2 and 40.4 % for indels and 21.2, 41.9 and 68.2 % for SNP, respectively. Amplicon-based WES on Ion Proton platform using HiQ chemistry emerged as a competitive approach, with improved accuracy in variants identification. False-positive variants remain an issue for the Ion Torrent technology, but our filtering strategy can be applied to reduce erroneous variants.

**Electronic supplementary material:**

The online version of this article (doi:10.1007/s00439-016-1656-8) contains supplementary material, which is available to authorized users.

## Background

Whole exome sequencing (WES) is a powerful method ideally designed to rapidly investigate all the coding sequences in human genome at base resolution, allowing to detect a wide spectrum of genetic variations (Adams et al. 2012; Wang et al. 2013; Samarakoon et al. 2014). Lowering costs of next generation sequencing (NGS) led to exponential increase of WES-based studies and this kind of approach has rapidly become the first-choice option to discover new disease genes in rare Mendelian disorders (Gilissen et al. 2011; Bamshad et al. 2011), as well as to evaluate risk alleles in complex disorders (Kiezun et al. 2012; Do et al. 2012). Recently, WES has been also increasingly applied in clinical and diagnostic settings (Yang et al. 2013; Biesecker and Green 2014; Lee et al. 2014), especially for cancer, pathologies with high genetic heterogeneity or in clinical cases where causative genes could not be clearly hypothesized. However, application of WES to clinical settings has some special requirements, such as increased sensitivity, full target sequence representation and the ability to rapidly perform sequencing with acceptable costs also for one or few samples (Dewey et al. 2014; Kim et al. 2015; Taylor et al. 2015). Increasing interest resulted in the development of several commercial exome enrichment products from different companies, such as Agilent, Nimblegen, Life Technologies and Illumina, mostly based on capture probes approach (Bodi et al. 2013; Chilamakuri et al. 2014). Similarly, several NGS sequencers based on different technologies are available to perform WES.h Sequence by synthesis with fluorescent reversible terminators from Illumina and semiconductor sequencing from Life Technologies are the most adopted solutions nowadays (Metzker 2009; Jünemann et al. 2013; Boland et al. 2013).

The semiconductor-based sequencing technology, launched in 2011 by Life Technologies (Rothberg et al. 2011; Merriman et al. 2012) and implemented in Ion Torrent NGS platforms, has emerged as an interesting alternative to Illumina-based sequencing, with the potential to be cost-effective and provide rapid turnaround time and greater flexibility in throughput. Indeed, the Ion Proton instrument, with 10-15 Gb output per run, enables investigators to study exomes, transcriptomes and custom target regions rapidly and at low cost (Jünemann et al. 2013; Boland et al. 2013). Several improvements have been recently delivered by Life Technologies for WES studies on the Ion Proton platform. In 2012 the company developed the AmpliSeq Exome kit, the first commercial method to perform target enrichment of the entire human exome by multiplex-PCR amplification, reducing time for library preparation. This method uses ultra-high multiplex-PCR approach based on the proprietary AmpliSeq technology to generate about 294,000 amplicons covering ~97 % of the bases in coding exons of human genes. In 2015, the company released the HiQ sequencing chemistry to improve accuracy of indel detection. Indeed, past comparisons of WES performed on Ion Proton and Illumina platforms revealed that the former performs with high accuracy at SNP discovery, but has a high ratio of false positives in the identification of small indels (Jünemann et al. 2013; Boland et al. 2013; Zhang et al. 2015). This posed serious challenges in downstream data analysis, considering that most work-flows search for variants that potentially alter gene function, particularly loss of function variants like indels and stop-gain mutations (Cooper and Shendure 2011; Isakov et al. 2013; Wang and Xing 2013). Since the vast majority of WES studies have been performed on Illumina sequencers, most technical optimization studies have focused on that particular platform (Chilamakuri et al. 2014; Head et al. 2014; van Dijk et al. 2014). Similarly, most bioinformatic methods are optimized for analysis of Illumina-based data (Hatem et al. 2013; Ross et al. 2013; Pabinger et al. 2013; Ghoneim et al. 2014; Yi et al. 2014; Laehnemann et al. 2015), while strategies to improve data analysis and variants identification on Ion Torrent platforms have not been discussed in detail so far.

Overall, an independent analysis of WES performance on Ion Proton sequencer using AmpliSeq Exome kit and the latest HiQ chemistry and a detailed comparison with Illumina-based results are still lacking, as well as alternative strategies for data analysis and filtering of false-positive variants.

Thus, we decided to perform a detailed technical evaluation of sequencing performances based on a dataset of 34 exomes produced using AmpliSeq Exome kit and Ion Proton platform. Moreover, we compared WES data from the NA12878 human reference sample obtained with v3 and HiQ chemistries to assess improvements in variants identification and characterize the properties of erroneous calls. This sample has been deeply characterized through time using multiple sequencing and genotyping platforms, and in 2013 Genome in a Bottle Consortium (GIAB), part of the National Institute of Standards and Technology (NIST), has distributed the first set of gold standard calls based on integration of 13 different datasets of this sample obtained using different NGS technologies (Zook et al. 2014). This constantly updated set of variants is now broadly accepted as a standard for variant identification benchmarking. By comparing our results with gold standard variants provided by GIAB (Zook et al. 2014), we analyzed in detail the performance of variants identification for both SNPs and indels and developed a filtering strategy to reduce false-positive calls.

## Materials and methods

### Comparison of target regions across different exome capture kits

The list of target regions included in the capture kits was obtained in BED file format from the vendor site for AmpliSeq Exome (Life Technologies), SeqCap EZ Exome v2/v3 (Roche) and SureSelect Human All Exon v5/v6 (Agilent). The full list of CDS coding exons from RefSeq was obtained from UCSC Genome Browser, corresponding to CCDS release 17 (CDS list). The list of clinically relevant genes was obtained from ClinVar database on 30 April 2015. We included in our analysis only genes reported as pathogenic, likely pathogenic, risk factors, or drug response and annotated with a gene symbol, for a total of 3399 genes (ClinVar list). Another list was created including the 56 genes indicated by the American College of Medical Genetics in the list of actionable genetic findings to be reported to patients when performing exome sequencing analysis (ACMG list) (Green et al. 2013). Coordinates of coding exons of the genes in each list were obtained in BED file format from the UCSC Genome Browser. Using BEDtools (Quinlan and Hall 2010), we compared each kit target regions with CDS, ClinVar and ACMG lists to determine the fraction of bases addressed and the number of genes that have at least one exon partially or fully not addressed.

### Exome sequencing

Exome sequencing was performed for 34 subjects using Ion Proton platform (Life Technologies) and AmpliSeq Exome kit (Life Technologies) for library preparation. Briefly, 100 ng of gDNA was used as starting material in the AmpliSeq Exome amplification step following manufacturer’s protocol. The final sequencing libraries were inspected and quantified using Bioanalyzer 2100 instrument and DNA HS kit (Agilent Technologies). All libraries were diluted to 100 pM working solutions and then pooled as needed to perform the template preparation on Ion OneTouch 2 (Life Technologies) according to manufacturer’s protocols. Of the reported 27 sequencing runs: the first 15, including 24 samples, were performed using Ion PI Template OT2 200 v2 and Ion PI Sequencing 200 v2 kits (Life Technologies); 5 runs, including 10 samples, were performed with v3 of the same kits; 5 runs, including 9 samples, were performed using Ion PI Hi-Q OT2 200 kit and Ion PI Hi-Q Sequencing 200 kit. Templated Ion Sphere Particles (ISP) were then enriched for positive ISP using Ion OneTouch ES (Life Technologies) and sequenced on Ion Proton sequencer using Ion PI chip v2 or v3 (Life Technologies), as reported in Supplementary table 7. Basecalling and sequence alignment were performed for all samples using Ion Torrent Suite software v.4.4.0.6 and genetic variants were then identified using Torrent Suite Variant Caller pipeline v.4.4.0.6 (TVC) with the optimized parameters provided by the manufacturer for AmpliSeq Exome.

The NA12878 reference gDNA was obtained from Coriell Cell Repositories and used to produce an AmpliSeq Exome library with the same procedure described above, except for library quantification that was performed using RealTime PCR and Ion Library TaqMan Quantification kit (Life Technologies). The same library was then sequenced twice with the v3 version of the Ion PI Template OT2 200 and Ion PI Sequencing 200 kits and one full Ion PI v2 chip; once using HiQ version of the kits and one full Ion PI v3 chip.

### Variants identification in public datasets

Aligned reads in BAM file format from eight independent exome sequencing experiments performed on NA12878 DNA using AmpliSeq Exome and the new HiQ sequencing chemistry were obtained from the Ion Community repository (https://ioncommunity.lifetechnologies.com/docs/DOC-9389). Genetic variants were then identified using TVC v.4.4.0.6 and the optimized parameters provided by the manufacturer for AmpliSeq Exome.

Exome sequencing data on the NA12878 using Illumina platform were obtained from 1000G repository (ftp://ftp-trace.ncbi.nih.gov/1000genomes/ftp/technical/working/20120117_ceu_trio_b37_decoy/). This BAM file was already modified for duplicate removal, realignment around indels and base quality score recalibration, according to GATK best practices. Genetic variants were identified using GATK HaplotypeCaller v3.2-2 (DePristo et al. 2011) with default parameters, but-stand-emit-call set to 20.

### Assessing coverage influence on variants identification

All the BAM files from the 15 non-HiQ samples with mean coverage above 90 were downsampled randomly using SAMtools (Li et al. 2009) to generate new alignments at 8 levels of coverage: 90, 80, 70, 60, 50, 40, 30 and 20× mean coverage. Depth and breadth of coverage over the AmpliSeq Exome target regions were calculated for all the 280 generated BAM files using BEDtools coverage function. Genetic variants identification was performed on each file using the command-line implementation of the TVC. In-house developed Perl/R scripts were used to compute distribution of variants and base coverage across various simulations and determine the relationship between coverage parameters and variants identification results.

To confirm the results from our simulations and verify that the same assumptions are validated for HiQ chemistry-based samples, we downsampled the nine BAM files of NA12878 sequenced with HiQ to 90 and 70× mean coverage, performed variants identification with the TVC and then compared results with those obtained using the full dataset, performing specificity and sensitivity tests as described below in “Study of performance” section.

### Analysis of hard-to-sequence regions in AmpliSeq Exome

We used BEDtools to generate a subset of the CDS list, including only exons present in the AmpliSeq Exome design, and calculated the breadth of coverage across the targeted coding exons for all our 34 sequenced samples. Comparing the coverage results, we identified the exons that are always absent from sequencing data (fraction of covered bases equal to zero) or always poorly covered, showing a fraction of covered bases ≤0.1, 0.25 or 0.5. Since the AmpliSeq Exome kit is composed by about 294,000 different PCR amplicons, we also characterized the coverage over each amplicon to identify those displaying poor sequencing results across different samples. Using BEDtools coverage and nuc functions, we calculated the amplicons mean coverage across all samples and studied the relationship between amplicons coverage and their GC content. For each amplicon we also calculated the proportion of reads that is captured in the overall dataset, expressed as the ratio between reads of the amplicon and million total reads. This will provide a better estimation of how different amplicons are represented in the sequencing experiments.

### Study of performances in variants identification for HiQ chemistry

To evaluate the performances of HiQ-based experiments in terms of detected variants, we compared our results with the set of reference variants identified in the NA12878 sample by GIAB consortium and provided as true variants dataset for benchmark (Zook et al. 2014). NIST v2.19 true variant calls in high confident regions were obtained from GIAB repository (ftp://ftp-trace.ncbi.nih.gov/giab/ftp/release/NA12878_HG001/NISTv2.19/) and used in our analysis.

We then compared variants identified by the consortium with those identified in our experiments performed using v3 chemistry and HiQ chemistry, as well as with variants identified in the HiQ datasets from Ion Community and in the Illumina dataset.

First of all, to ensure the best uniformity in variants representation, all VCF files were normalized by splitting variants with multiple variant alleles and left aligning indels. We then restricted the analyzed variants to those falling in the high confident region as determined by GIAB and located within the AmpliSeq Exome target regions. Finally, since we were interested in performance on coding regions, we limited the considered variants to those located in the CDS regions plus three flanking base pairs.

Indel and SNP variants from each dataset were then compared separately with the GIAB reference calls to identify false positives, true positives and false negatives. We also compared false positive, true positive and false negative variants across the nine different HiQ datasets to determine systematic errors and reproducibility of true calls.

### Characterization of errors in variant calling

To better evaluate erroneous calls and dissect possible sources of error, we studied several parameters in false-positive and false-negative calls for both indel and SNP variants identified using HiQ chemistry. In details, for false-positive variants we analyzed the distribution of 11 parameters calculated by the TVC and reported in the VCF files: alternate allele observation (AO), read depth (DP), flow-space alternate allele observations (FAO), flow space read depth (FDP), flow evaluator failed reads ratio (FXX), genotype quality (GQ), length of homopolymer (HRUN), quality per read length (QD), variant quality (QUAL), strand bias ratio (STB) and strand bias *p* value (STBP). Moreover, we determined which proportion of these variants was present as multiallelic variant calls in the original VCF file. For false-negative variants, where TVC information is missing, we evaluated the proportion of missed calls due to low read depth (<10 reads) and indels calls were also inspected to identify possible recurrent motifs. Moreover, for both false-positive and false-negative indels we also considered indels length distribution. Finally, we used Perl scripts to intersect the dataset of false positives, false negatives and true positives from single experiments to evaluate if sequencing errors would be recurrent across different samples. For this analysis, we matched only variants that recur identical across the nine HiQ dataset, namely variants presenting the same position as well as identical reference and alternate alleles.

### Determining filtering strategy for indel and SNP false-positive variants

Based on the distribution of parameters described above, we selected five that could better discriminate false-positive from true-positive variants and generated ROC curves, separately for SNP and indels. For SNPs we used FAO, FDP, GQ, QUAL, STB; for indels: FAO, FDP, GQ, HRUN, QUAL. Using the eight datasets from Ion Community as training set we studied the effect of various combinations of these five parameters and created a false-positive filter with three different threshold of retained true-positive calls: high, medium and low-stringency filters which would retain >90, >95 and >99 % of true positives, respectively (Table 3). Moreover, we evaluated additional strategies to rank the robustness of identified indels based on their length and occurrence as multiallelic variants.

The filtering settings we defined were then applied on the HiQ data produced in our laboratory to test if this strategy could produce the desired results on samples not belonging to the training set.

## Results

### Characterization of AmpliSeq Exome and analysis of target regions

**Fig. 1:**
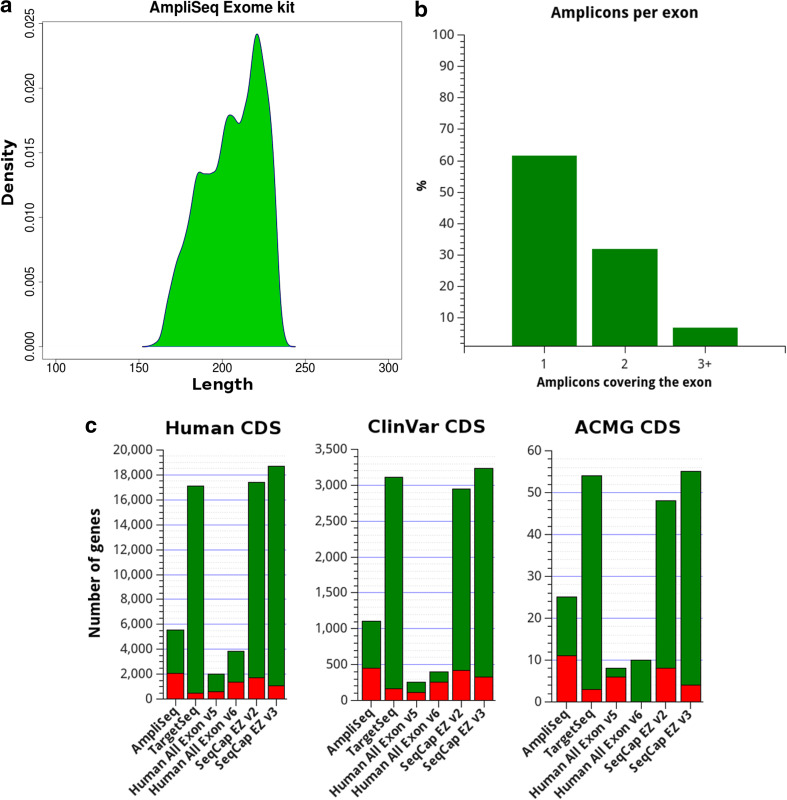
AmpliSeq exome properties and target region analysis. **a** Length density distribution calculated from the dimension of amplicons generated with the AmpliSeq Exome kit as determined from the provided BED. In the kit design, most of the CDS exons are covered by a single amplicon (**b**). The comparison of target regions across 6 different enrichment kits (**c**) revealed that no one fully address all the human CDS nor relevant clinical genes. The *bar graph* represents the number of genes with at least one exon partially addressed (*green*) or completely missed (*red*). Compared exome capture kits include: AmpliSeq Exome and TargetSeq Exome (Life Technologies), SureSelect Human All Exon v5/v6 (Agilent Technologies) and SeqCap EZ v2/v3 (Roche)

The AmpliSeq Exome kit (Life Technologies) uses a multiplex-PCR approach to simultaneously generate about 294,000 amplicons designed on CDS sequences of the human genome (Table 1). Size of amplicons ranges between 156 and 240 bp (Fig. 1a) and the kit design addresses 300,887 CDS exons, 61.5 % of whom are covered by a single amplicon (Fig. 1b). To better determine the actual content of target regions and the extent of non-addressed clinically relevant regions, we compared the target intervals of AmpliSeq Exome kit with those of other popular exome capture kits, namely Life Technologies TargetSeq, Agilent SureSelect and Roche SeqCap EZ Exome. As described in methods, we determined for each kit the fraction of CDS bases comprised in the design and extrapolated the number of genes not fully addressed, with particular interest on ClinVar pathological genes and the 56 genes included in the incidental findings recommended report by ACMG. AmpliSeq Exome target regions covered a high proportion of bases in human CDS (97.5 %), ClinVar genes (94.1 %) and ACMG genes (98.9 %). However, even this small fraction of missed bases resulted in several genes with at least one exon completely missed: 2041 (7 %), 449 (13 %) and 11 (19 %) genes show this issue in human CDS, ClinVar and ACMG gene list, respectively (Fig. 1c). An example of a gene not completely addressed is reported in Supplementary figure 1a. Detailed statistics calculated for each exome enrichment kit are reported in supplementary file 1 and the complete list of the exons not fully addressed is provided in supplementary tables 1–6.

**Table 1:** AmpliSeq exome kit main properties

Amplicons	Mean amplicons per pool	Amplicons length (mean/min/max)	Target region size (Mb)	CDS bases included	CDS exons addressed
293,903	24,492	206/156/240	57.74	97.5 %	298,901

### Sequencing results on Ion Proton platform

In our dataset of 27 sequencing experiments on Ion Proton platform the final throughput obtained from a single PI chip varies from 6 up to almost 18 Gb, with a mean throughput of 9.3 and 11.5 Gb using PI v2 chip and chemistry v2 and v3, respectively. Sequencing runs using PI v3 chip and HiQ chemistry resulted in 16.9 Gb mean throughput (supplementary table 7). The whole protocol, from library preparation to sequence production, was completed in 48–60 h. Detailed results of exome sequencing on the 34 samples included in this study are reported in supplementary table 8. Mean coverage per sample was 42–148× and mapped reads were 21.7–53.9 M. AmpliSeq Exome enrichment protocol resulted in >85 % on-target reads for all samples except S25, with a percentage of target bases covered at least 20× of 72.1–94.4 %. Uniformity of coverage, defined as the percentage of bases in target regions with a read depth at least 20 % of the average sample coverage, is >80 % for all samples except S4 and S6. Coverage uniformity is confirmed also by inter-quartile range of base coverage (IQR), as reported in supplementary table 8. These data resulted in a number of identified variants between 42,555 and 54,127 using the Torrent Variant Caller (TCV) with the optimized parameters provided by the manufacturer (see “Materials and methods”).

### Coverage across target regions and characterization of problematic regions

Given a correct read length distribution of input library, the AmpliSeq Exome method showed a high uniformity of coverage across all samples (supplementary table 8). The fraction of target exons fully covered is >90 % in all samples except sample S4, with a mean of 96.8 %, even for samples with mean coverage as low as 40× (Supplementary figure 2a).

We then analyzed the coverage obtained per single amplicon to identify those that are hard to be sequenced and possible factors influencing sequencing performance. Mean coverage per amplicon ranges from 0.03 to 8886, with each amplicon capturing a fraction of the total throughput from 0.01 to 167 reads/million reads (Supplementary figure 2b). Looking at the distribution of % GC across amplicons, there are 5458 and 2156 amplicons with  % GC >75 (high GC) and <25 (low GC), respectively (Supplementary figure 2c). Amplicons with high GC content have a median coverage of 14.2 and 41.8 % have mean coverage <10, while amplicons with low GC content have a median coverage of 114 and only 1.4 % have mean coverage <10. We found 3691 amplicons (1.2 %) with a mean coverage <10 across all the sequenced samples, each one represented by <0.2 reads/million reads. Of these amplicons 2282 (62 %) are characterized by high GC content (>75 %). Instead, amplicons with GC content <25 % did not show a particular coverage bias and they represent only 31 (0.8 %) of the low covered amplicons (Supplementary figure 2d).

Besides high GC content, other factors influence sequence coverage. Detailed analysis of the sequences included in target regions and affected by coverage issues, revealed that some of them are consistent across all samples. In detail, 509 CDS exons resulted always covered by less than 10 reads; 51, 15 and 7 CDS exons resulted always partially sequenced with <50, 25 and 10 % of their sequence addressed, respectively. Moreover, in seven exons we found portions always missed by sequencing. This resulted in 515 protein coding genes containing hard-to-sequence regions, including also 90 known pathogenic genes reported in ClinVar. An example is reported in Supplementary figure 1b. The complete list of exons hard to be sequenced is reported in supplementary table 9.

### Coverage effects on variant calling

In each sample the number of variants identified is, as expected, strongly influenced by coverage metrics, particularly by the fraction of bases covered at least 20× (Supplementary figure 3). Downsampling approach allowed us to better estimate the relationship between coverage metrics and performances in variant calling. Our analysis showed that, given a library with correct read length distribution, in each sample the fraction of bases covered at least 20× is exponentially related to the mean coverage (*r* ^2^ = 0.974), with a marked decline below 50× and a maximum reached above 120× mean coverage (Supplementary figure 4a). Analysis of the fraction of variants lost at various downsampling steps showed a linear relationship (*r* ^2^ = 0.986) with the fraction of bases covered at least 20× (Supplementary figure 4b).

We then calculated the expected performance for a sample sequenced at 130×, 90× and 70× mean coverage. That would correspond to an experimental design with 2, 3 or 4 exomes sequenced on a single PI chip v3, considering an optimal throughput of 16 Gb. Based on the generated distributions, a sample covered 130× or more will result in >92 % bases covered at least 20× and ensures maximum performance in variants detection. Downsampling to 90× and 70× produces 90.1 and 87.2 % bases covered >20× resulting in 3.2 and 4.5 % loss of variants, respectively (Supplementary figure 4). These estimations were confirmed by downsampling to 90 and 70× mean coverage the nine NA12878 datasets generated on Ion Proton platform. Overall, 96.4–98.7 and 94.9–97.6 % of total variants were retained when downsampling to 90 and 70×, respectively. To better evaluate the impact of downsampling we also calculated the concordance with the gold standard GIAB calls as described in methods. After downsampling to 90×, we detected 0.9–5.5 and 0.5–1.4 % loss in true-positive variants for indels and SNPs, respectively. Instead, downsampling to 70× resulted in 4.3–8.8 and 1.1–2.2 % loss in true-positive variants for indels and SNPs, respectively (Supplementary figure 5).

### Estimation of accuracy in variants identification

**Fig. 2:**
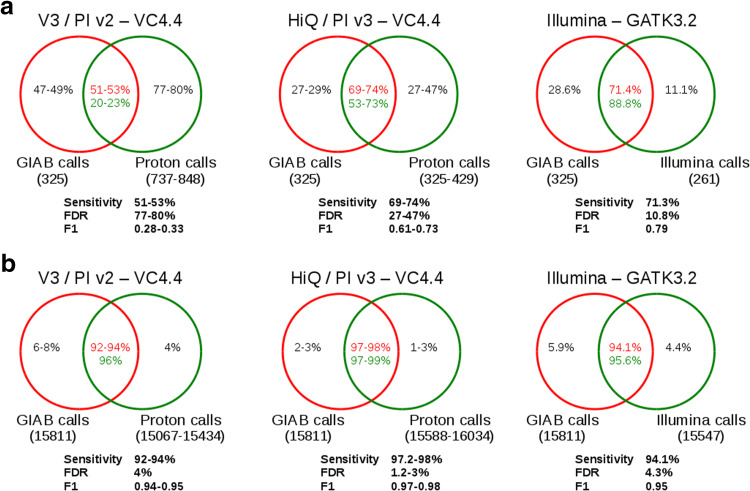
Accuracy of variant identification. The performance of variant identification was assessed separately for indel (**a**) and SNP variants (**b**) by comparing the variants identified on the Ion Proton platform with the gold standard calls provided by GIAB consortium for sample NA12878. The comparison was conducted on samples sequenced using the v3 chemistry and the PI v2 chip, as well as the HiQ chemistry and the PI v3 chip. Results were also compared with those obtained using an Illumina-based exome sequencing of NA12878. The total number of variants in the GIAB and tested datasets are reported in *brackets* and the % of variants overlapping or unique to each compared dataset are shown in the *Venn diagrams*. Accuracy statistics are also reported for each comparison

We estimated the accuracy of variants identification for the NA12878 reference sample by comparing the datasets of variants identified by Ion Proton platform (Table 2) with the set of gold standard variants reported by GIAB consortium. The dataset of variants from GIAB addressed by AmpliSeq Exome kit within CDS sequences was composed by 325 indels and 15,811 SNPs. The dataset produced by Ion Proton platform accounted for ~790/370 indels and ~15,250/15,800 SNPs for v3 and HiQ chemistry data, respectively. Data produced with HiQ chemistry and PI v3 chip showed a significant increase in accuracy compared to those produced using v3 chemistry and PI v2 chip. Results for SNP variants are now similar, with slightly better sensitivity, to those obtained from the Illumina dataset, while false-positive indels remain markedly higher in the HiQ dataset with and FDR value up to 47 % (Fig. 2). Detailed results on variants identification performances for each dataset are reported in Supplementary table 10.

**Table 2:** NA12878 exome datasets used in the study

Sample	Source	Chemistry/chip	Gb	Mean coverage	% of bases covered ≥20X	Variants	Mapped reads
NA12878_1	Local	v3/v2	8.4	123	89.7	52,429	49,404,723
NA12878_2	Local	v3/v2	10.9	160	91.6	52,820	70,938,267
NA12878_HiQ	Local	HiQ/v3	14.2	211	96.9	55,289	82,542,306
HiQ_012	Ion community	HiQ/v3	7.2	125	94.7	53,703	42,829,518
HiQ_018	Ion community	HiQ/v3	7.3	126	94.9	53,367	41,924,486
HiQ_022	Ion community	HiQ/v3	9.7	168	96.5	53,721	54,412,206
HiQ_029	Ion community	HiQ/v3	8.8	152	95.5	53,691	49,108,758
HiQ_030	Ion community	HiQ/v3	8.8	152	95.5	53,640	48,942,525
HiQ_036	Ion community	HiQ/v3	6.7	116	94.4	53,655	39,157,852
HiQ_042	Ion community	HiQ/v3	8.2	142	95.9	53,594	45,781,947
HiQ_046	Ion community	HiQ/v3	8.7	151	96.2	53,651	48,364,571

The table reports the results of the 11 exome sequencing experiments on the NA12878 sample used in the study. The source of the data is indicated as Local, when specifically produced for this study, or Ion community, when downloaded from public repository

### Analysis of false-positive and false-negative variants in the HiQ datasets

**Fig. 3:**
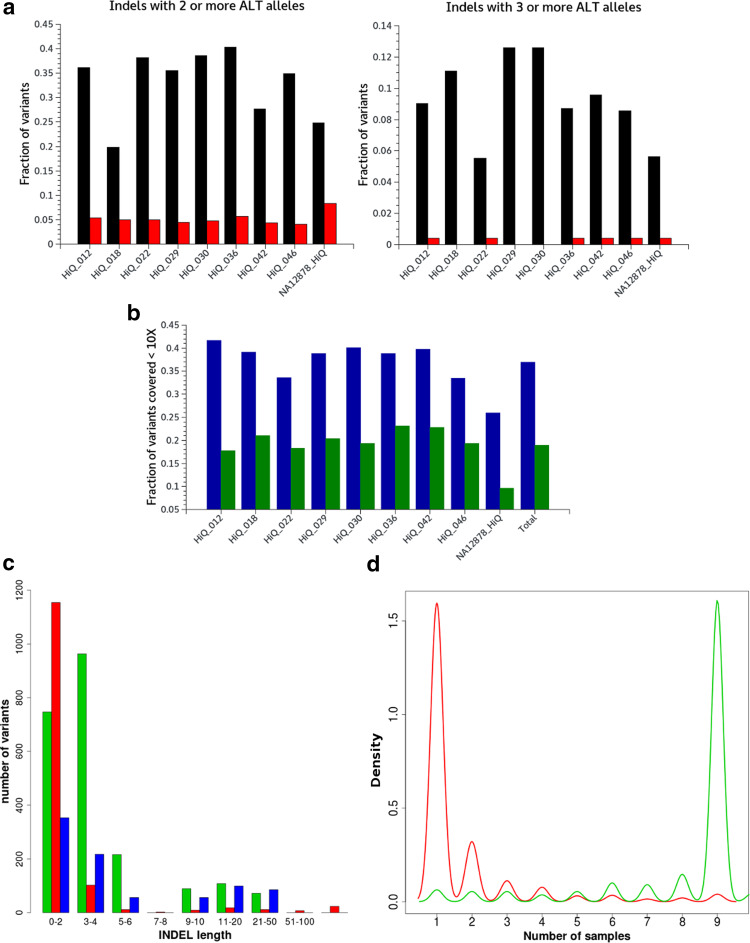
Analysis of variant calling errors in HiQ datasets. A detailed characterization of errors in variants identified by the TVC using the optimize parameters provided by the manufacturer. **a** Fraction of true-positive (*red*) and false-positive (*black*) variants occurring with more than 2 or more than 3 alternate alleles in the corresponding VCF file. **b** Fraction of false-negative SNP (*blue*) and indels (*green*) with read depth <10 in the corresponding sample. The indel length of false positive (*red*), true positive (*green*) and false negative (*blue*) calls identified across the nine HiQ datasets is analyzed in **c**. True positive calls are highly consistent across the nine HiQ samples, while false-positive calls are often run specific as suggested by the density plot (**d**), that evidences the recurrence of false positives (*red*) and true positives (*green*)

**Fig. 4:**
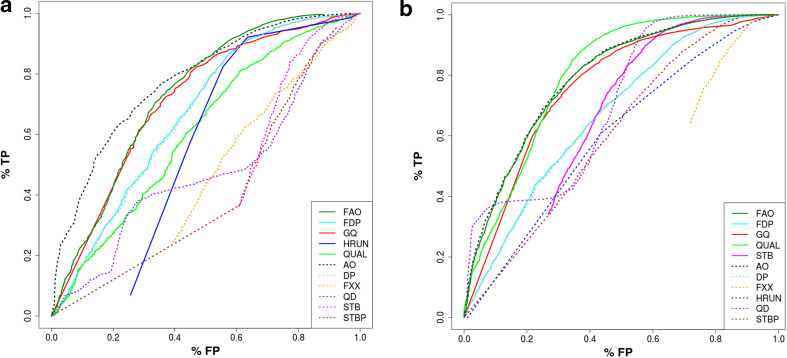
Study of parameters for variant filtering. We evaluated the distribution of 11 parameters reported by the TVC for indel (**a**) and SNP (**b**) variants identified from the HiQ datasets. *AO* alternate allele observation, *DP* read depth, *FAO* flow-space alternate allele observations, *FDP* flow space read depth, *FXX* flow evaluator failed reads ratio, *GQ* genotype quality, *HRUN* length of homopolymer, *QD* quality per read length, *QUAL* variant quality, *STB* strand bias ratio, *STBP* strand bias *p* value. The five parameters selected for filtering are reported as *solid lines*

To investigate the nature of errors in variants identification on the Ion Proton platform, we performed a detailed characterization of both false-positive and false-negative variants described above. We first evaluated the distribution of 11 parameters reported by the Torrent Variant Caller (see “Materials and methods”) across false-positive and true-positive variants for indels and SNPs separately (Fig. 4). Moreover, we assessed the proportion of false-positive and true-positive variants represented as multiallelic variant calls in the original VCF file. Concerning indels, variants occurring with three or more alternate alleles represented 8.5–12.5 % of false positives and only 0–0.4 % of true positives (Fig. 3a). No significant differences were detected for SNP variants (data not shown). For false-negative variants, we evaluated the proportion of missed calls due to low read depth (<10 reads), showing that 26–41 % of false-negative SNPs and 10–23 % of false-negative indels are due to low coverage (Fig. 3b). Further inspection of the false-negative calls with read depth >10× revealed that triplet repetition and homopolymeric regions are recurrent among missed variants (data not shown). Analysis of the indels length showed that most false positives and false negatives are represented by short (1–2 bp) insertion/deletions, while large ones above 100 bp are almost all erroneous calls (Fig. 3c). We then compared read length distribution and variant identification performances in the nine HiQ datasets. The NA12878_HiQ dataset, that showed lower performances in variant identification (see Supplementary table 10), revealed a substantial deviation from the expected distribution with a loss of long fragments (Supplementary figure 6).

Finally, we analyzed the recurrence of false-positive and true-positive variants across the nine HiQ datasets. True-positive variants were consistent through the datasets and 81 % were found in at least 8 samples, while false-positive variants resulted extremely variable and reported only in a single dataset in 71 % of cases, with only 6 % recurring in more than half samples (Fig. 3d).

### Estimation of filtering parameters in the HiQ datasets

Using the distributions of variant quality parameters estimated above, we selected five parameters that better discriminated true positives from false positives and we defined a set of thresholds that could be used to filter out false-positive calls (Fig. 4). For SNPs and indels separately we used the eight HiQ datasets from Ion Community to identify three sets of parameters corresponding to three levels of filtering, namely high, medium and low-stringency filters, that should retain 90, 95 and 99 % of true positive calls, respectively (Table 3). By applying these filters to NA12878_HiQ dataset, we obtained the predicted level of true positives and reduced the amount of false positives by 10.2, 21.2 and 40.4 % for indels and 21.2, 41.9, 68.2 % for SNPs (Table 4).

**Table 3:** Setting of filtering parameters

Stringency	% TP retained	GQ < *x*	FDP < *x*	QUAL < *x*	HRUN > *x*	FAO < *x*
**INDELs**						
Low	99	5	10	20	6	4
Medium	95	8	20	30	5	4
High	90	10	25	40	4	4

Stringency	% TP retained	GQ < *x*	FDP < *x*	QUAL < *x*	STB > *x*	FAO < *x*
**SNPs**						
Low	99	5	6	20	0.90	2
Medium	95	10	6	20	0.70	2
High	90	15	10	30	0.60	2

The settings of filtering parameters for low, medium and high-stringency filters are reported together with the % of true-positive calls (TP) retained using each filter

**Table 4:** Test of proposed filtering settings on NA12878 dataset

Stringency	SNPs		INDELs	
	% TP retained	% FP filtered	% TP retained	% FP filtered
Low	99.2	21.2	98.9	10.2
Medium	96.1	41.9	95.1	21.2
High	89.9	68.2	89.8	40.4

The table reports the % of true-positive (TP) and false-positive (FP) variants retained applying each filter on the NA12878_HiQ dataset

## Discussion

### Characteristics and sequencing performances of the AmpliSeq Exome kit

The AmpliSeq Exome kit (Life Technologies) uses a multiplex-PCR approach to simultaneously generate about 294,000 amplicons covering most of the CDS sequences in the human genome (Table 1; Fig. 1). The entire sequencing process, from library preparation to variant identification, can be completed in 48 h, thus providing a rapid method for exome investigation. The AmpliSeq Exome protocol on the Ion Proton platform showed robust sequencing results and the recently introduced HiQ chemistry and PI v3 chip have improved the sequencing throughput to 16–17 Gb per run. The use of a PCR-based library preparation approach provides great specificity in target sequence enrichment, as indicated by the percentage of on-target reads usually around 90 %. This should also minimize the waste of sequencing throughput due to off-target data, enhancing the productivity of the system. The analysis of coverage across the sequenced samples revealed high coverage uniformity, with most of the targeted bases represented in sequencing results also at lower mean coverage (Supplementary figure 2a, b). The analysis of the target regions addressed by AmpliSeq Exome kit revealed that it includes most of the bases present in human CDS sequences (~97 %). However, we found that up to 13 % of pathogenic genes from ClinVar could not be fully analyzed, a data that should be considered for diagnostic applications. This could be due to updated RefSeq annotations resulting in new exons placement or difficulties in amplicon design for certain genomic regions (see an example in Supplementary figure 1). The inability to fully capture the entire exome is a known issue (Bodi et al. 2013; Chilamakuri et al. 2014; Meienberg et al. 2015) and comparison among the main exome enrichment kits indicates SureSelect Human All Exon (Agilent Technologies) as the best one, particularly in terms of representation of medically relevant genes.

In the AmpliSeq Exome kit design most of the exons are addressed by one or two amplicons (Fig. 1b), thus the sequencing performance of each amplicon must be optimal to avoid loss of information on specific exons. One of the main factors influencing PCR amplification is the GC content of the amplified region and this parameter is also critical for NGS sequencing performance on both Illumina and Ion Torrent platforms (Liu et al. 2012; Quail et al. 2012; Ross et al. 2013). In our analysis, amplicons with a % GC content above 75 tend to fail in sequencing, resulting in a mean coverage below 10 across the analyzed samples (Supplementary figure 2c, d). Instead, contrasting with previous studies on Ion Torrent PGM bacterial sequencing (Quail et al. 2012; Ross et al. 2013), we did not measure a significant reduction in sequencing performances for amplicons with low GC content. Our detailed analysis of the sequences included in the target regions, but affected by coverage issues, revealed that some of them are consistent across all samples, with 515 protein coding genes containing hard-to-sequence regions. These genes include also 90 known genes reported as pathogenic, likely pathogenic or risk factors in ClinVar. The described issues on coverage and target regions confirm that is hard to obtain a complete picture of clinically-relevant genomic alterations when using exome sequencing solutions designed for research purposes (Meienberg et al. 2015). The high fraction of exons from ClinVar and ACMG genes completely missed by AmpliSeq Exome approach (Fig. 1c) suggests avoiding its use for clinical applications.

### Accuracy in variants identification

Coverage metrics are one of the main factors influencing variant calling process (Hou et al. 2013; Sims et al. 2014; Kim et al. 2015), as also seen in our study. Our downsampling analysis showed that, given an optimal sequencing library, a maximum in variants identification performance is reached above 120× mean coverage and that about 99 and 95 % of true variants could be identified at 90 and 70× mean coverage, respectively. Based on this analysis, three samples can be sequenced on a single PI v3 chip with high performance in variant calling, or up to four samples can be pooled together if sample size and not full detection of variants is critical for the study, as in genotyping approaches aggregating data from multiple samples for group comparisons.

To estimate the actual accuracy in variant identification provided by an AmpliSeq Exome sequencing experiment, we decided to compare our results on the NA12878 human reference sample with the latest version of the gold standard variant calls provided by GIAB Consortium (Zook et al. 2014), a set of variants now broadly accepted as a standard for variant identification benchmarking. This analysis revealed a significant improvement from v3 chemistry to HiQ chemistry (Fig. 2), with F1 value increasing from ~0.3 to ~0.65 and from ~0.94 to ~0.97 for indel and SNP variants, respectively. Previous studies comparing sequencing technologies have shown that variants identification is particularly difficult for indels on Ion Torrent data, with a high level of false-negative and false-positive calls (Jünemann et al. 2013; Boland et al. 2013). The HiQ chemistry datasets showed variant calling performance comparable with those obtained from the Illumina-based exome sequencing (Fig. 2) in terms of sensitivity. However, the Ion Proton platform confirmed to have more difficulties with indel calls and still produces a higher number of false-positive indels, with an FDR of ~35 %, significantly higher than the Illumina FDR of ~10 %.

The accuracy of variants identification was also strongly influenced by the read length profile of single experiment, with the loss of long fragments being the main issue. This could be explained by amplicon-based design of AmpliSeq Exome. Sequences produced from forward and reverse strands of each amplicon start at 3′ and 5′ ends of the addressed exon, so that shorter reads would result in low coverage at its center.

Overall, we also noted that the sensitivity for indel variants identification is ~70 % for both platforms, suggesting that a large fraction of actual indels would be missed in a standard exome sequencing experiment. This confirms previous findings reporting that specialized approaches in both sequencing and data analysis are required to effectively address this kind of variants.

### Characterization of sequencing errors and filtering strategies

Given the increased sensitivity when using HiQ chemistry, the main bias affecting variants identified from AmpliSeq Exome sequencing experiment is the high proportion of false-positive calls. To address this issue, we analyzed in detail false-positive (FP), false-negative (FN) and true-positive (TP) calls and developed a filtering strategy to effectively reduce the proportion of false positives. First of all, we analyzed for false-positive and true-positive variants the distribution of 11 variant quality parameters calculated by the Torrent Variant Caller. For both SNP and indel calls, we selected five parameters that could better discriminate erroneous calls (Fig. 4) and used them to develop a set of filters to remove false-positive calls. Depending on the desired level of sensitivity, high, medium or low-stringency filters remove up to 68 and 40 % of false-positive SNPs and indels, respectively (see Tables 3, 4). The medium-stringency filter should be the best choice when analyzing single samples, as it retains more than 95 % TP variants and ensures a relevant reduction of FP variants that could confound subsequent variants interpretation. The low-stringency filter is recommended when considering pedigree data, where maximum sensitivity may be preferred to search for causative mutations and most of FP variants could be filtered out by segregation analysis. Finally, the high-stringency filter could be useful in large sequencing projects where the main focus is the global estimation of allele frequencies. In this case, the maximum reduction in FP variants could greatly enhance association analysis results, while the moderate loss of TP variants on single samples could be compensated by the large sample size.

Our analysis also revealed other peculiar aspects of erroneous indel calls, the major class of variant error reported for the Ion Torrent technology. Even if they may not be used for filtering, they could be used to prioritize indel variants in downstream analysis. As previously described for the Ion Torrent sequencing (Liu et al. 2012; Quail et al. 2012), also in our HiQ datasets most of the false-positive indels were short insertions/deletions of 1–2 bp (Fig. 3c). False-positive indels tend to occur with multiple alternate alleles in VCF files, mainly due to short homopolymer or triplet repeats erroneously identified as multiple indel alleles with different lengths. Particularly, indels presenting three or more alternate alleles have a high probability to be false positives. Concerning false-negative calls in our datasets, they are only partially explained by coverage issues (Fig. 3b). Triplet repetitions and homopolymeric regions are recurrent among missed variants, confirming that these kinds of variants are difficult to be detected using NGS technologies (Allhoff et al. 2013).

Finally, we analyzed recurrence of false-positive and true-positive variants across the nine HiQ datasets. True-positive variants showed to be highly consistent through the datasets, while false-positive variants resulted extremely variable and mostly reported only in a single dataset (Fig. 3d). This supports the hypothesis that false-positive calls, especially indels, are mainly represented by random errors associated to the high ratio of indels/read produced by the Ion Torrent sequencing technology (Bragg et al. 2013). The run-specific nature of false positives also suggests that an experimental design based on sequencing replicates of the same library could be an effective strategy to improve variant identification and filter out false variants.

## Conclusions

Amplicon-based exome sequencing on the Ion Proton platform provides rapid and cost-effective exome sequencing on human samples. Improved accuracy and sequencing throughput thanks to the latest HiQ chemistry and PI v3 chip provide good flexibility in experimental design, increasing productivity up to four samples per run, while maintaining acceptable performance in terms of variants identification. However, as in exome enrichment products from main competitors, the full exome cannot be completely addressed by AmpliSeq Exome kit, due to limitations in both target region PCR amplification and sequencing performance on specific genomic regions. Researchers should carefully consider the best exome enrichment kit based on their region of interest and here we provide a useful guide to assess missed exons in AmpliSeq Exome kit (Supplementary tables 1 and 9). Even if false-positive variants remain major issues in variant calling for the Ion Torrent technology, our analysis provides a useful filtering strategy to reduce their number. We identified a set of filters and peculiar properties characterizing false-positive variants that could be used together to significantly enhance performance in exome variants analysis.

## Availability of supporting data

The datasets supporting the results of this article are available after free registration in the Ion Community repository, https://ioncommunity.lifetechnologies.com/docs/DOC-9389.

The dataset for exome sequencing on the NA12878 using Illumina platform is available from 1000G repository, ftp://ftp-trace.ncbi.nih.gov/1000genomes/ftp/technical/working/20120117_ceu_trio_b37_decoy/.

Gold standard calls and high confident regions from GIAB used in this study are available in the NCBI repository, ftp://ftp-trace.ncbi.nih.gov/giab/ftp/release/NA12878_HG001/NISTv2.19/.

## Electronic supplementary material

Below is the link to the electronic supplementary material.

Detailed results of the comparison of exome capture kits assessed in the study (XLS 9 kb)

Supplementary tables 1–6. Lists of CDS regions not fully addressed in the 6 exome enrichment kits compared in the study. Gene symbol and ClinVar annotation are also reported (XLS 34183 kb)

Supplementary tables 7–8. Detailed results of the 27 sequencing runs and 34 exome sequencing data analyzed in the study (PDF 40 kb)

Supplementary table 9. List of regions within human CDS exons that emerged as hard to sequence intervals based on our analysis. Gene symbols and ClinVar annotations are also reported (XLS 127 kb)

Supplementary table 10. Detailed results on variant identification performances on the NA12878 sample, determined by comparison of WES datasets with gold standard variants provided by the GIAB consortium (XLS 11 kb)

Supplementary figures cited in the paper with figure legends (PDF 1661 kb)
