# Supplementary material for: Amplicon-based semiconductor sequencing of human exomes: performance evaluation and optimization strategies
Source: Hum Genet. 2016 Mar 22;135(5):499–511. doi: 10.1007/s00439-016-1656-8 (PMC4835520; doi:10.1007/s00439-016-1656-8)
Supplement: Supplementary file 1 [file 439_2016_1656_MOESM3_ESM.pdf]

Supplementary table 7. Sequencing throughput

Run	Chip loading (%)	Usable reads (M)	Samples	Throughput (Gb)	Sequencing Chemistry	Chip version
Run1_2013	79	68.2	1	9	v2	v2
Run2_2013	76	53.7	2	8.2	v2	v2
Run3_2013	91	82.9	2	12.3	v2	v2
Run4_2013	88	70.6	3	11.5	v2	v2
Run5_2013	86	71.3	3	11.1	v2	v2
Run6_2013	78	71.3	2	12.1	v2	v2
Run7_2013	65	48.1	2	7.9	v2	v2
Run8_2013	58	42.6	2	6.1	v2	v2
Run9_2013	64	52.5	4	8.6	v2	v2
Run10_2013	63	51	4	7.8	v2	v2
Run11_2014	75	65.3	3	9.9	v2	v2
Run12_2014	73	51.1	2	7.4	v2	v2
Run13_2014	75	58.5	2	9.9	v2	v2
Run14_2014	74	60.1	2	9.9	v2	v2
Run15_2014	75	53	2	8.2	v2	v2
Run16_2014	88	76	2	12.6	v3	v2
Run17_2014	81	75.6	2	10.3	v3	v2
Run18_2014	81	71.5	2	10.1	v3	v2
Run19_2014	86	76.6	2	12.1	v3	v2
Run20_2014	85	88	2	13.8	v3	v2
Run21_2014	82	68.4	2	10.6	v3	v2
Run22_2015	84	73.8	1	10.9	v3	v2
Run23_2015	90	93.2	1	16.6	HiQ	v3
Run24_2015	88	94.1	2	17.7	HiQ	v3
Run25_2015	91	95.6	2	17.9	HiQ	v3
Run26_2015	88	93.4	2	16.6	HiQ	v3
Run27_2015	85	86.8	2	16.1	HiQ	v3

Supplementary table 8. Sequencing results for each sample

Sample	Gb	Mean coverage	% of bases covered ≥ 20X	Variants	Mapped reads	On target reads (%)	Coverage uniformity (%)	IQR
S1	9.2	148	94.4	52882	53895205	92.9	91.8	128
S2	5.1	81	87.2	51835	29600331	91.7	89.5	72
S3	5.7	87	84.8	50017	36717647	86.3	86.7	85
S4	4.6	73	72.1	42555	29670536	90.5	76.1	93
S5	7.7	117	90.0	51714	49810202	86.0	88.6	108
S6	5.7	88	76.6	44370	36600843	89.9	78.0	108
S7	5.6	85	79.9	48782	35917358	85.5	82.3	89
S8	5.9	91	87.4	50724	37579354	87.5	88.6	85
S9	5.7	87	88.1	51092	41837419	85.7	89.7	77
S10	5.2	81	86.2	49919	38212766	89.7	88.6	75
S11	5.1	78	83.0	49791	36876134	88.7	86.9	74
S12	5.8	92	90.5	51795	33823693	93.1	91.3	80
S13	7.5	118	93.6	54127	51125018	87.2	92.4	99
S14	5.4	85	88.7	50087	31817461	91.7	90.2	76
S15	2.7	42	74.5	48677	18026501	86.8	91.4	37
S16	3.3	51	81.7	49376	21724373	87.2	92.1	43
S17	7.6	117	90.1	51578	48782010	87.7	88.4	112
S18	4.3	67	78.3	48137	13299662	88.6	85.9	63
S19	8.3	131	92.1	52126	50148863	90.1	90.2	116
S20	7.6	122	92.8	53154	47904878	91.0	91.4	105
S21	5.9	92	92.1	53415	40903034	86.4	92.8	76
S22	8.3	130	94.0	53586	52623486	89.0	92.1	110
S23	3.4	53	83.6	50378	23254998	86.5	92.4	44
S24	6.1	94	90.8	52419	36958853	88.4	91.4	79
S25	3.9	58	85.0	51096	27325623	83.1	92.2	48
S26	3.8	60	84.2	50337	25188336	87.8	91.6	51
S27	4.7	74	86.4	51034	27835836	90.5	89.9	67
S28	7.2	114	92.1	52073	43770847	90.7	91.1	98
S29	3.6	59	78.1	48097	23444306	92.4	87.9	55
S30	4.4	71	82.1	48427	28066337	93.4	87.7	71
S31	5.2	82	86.0	51218	34902958	89.5	89.4	77
S32	6.9	107	93.0	52597	45664724	87.4	94.1	90
S33	6.9	106	91.2	51414	46264665	88.4	90.6	93
S34	6.0	95	90.2	51444	39228833	88.5	92.6	84

Coverage uniformity indicates the % of bases in the target regions with a read depth at least 20 % of the average sample coverage. IQR report Inter-Quartile Range of base coverage.
