# Supplementary material for: Amplicon-based semiconductor sequencing of human exomes: performance evaluation and optimization strategies
Source: Hum Genet. 2016 Mar 22;135(5):499–511. doi: 10.1007/s00439-016-1656-8 (PMC4835520; doi:10.1007/s00439-016-1656-8)
Supplement: Supplementary file 2 [file 439_2016_1656_MOESM6_ESM.pdf]

Supplementary figure 1

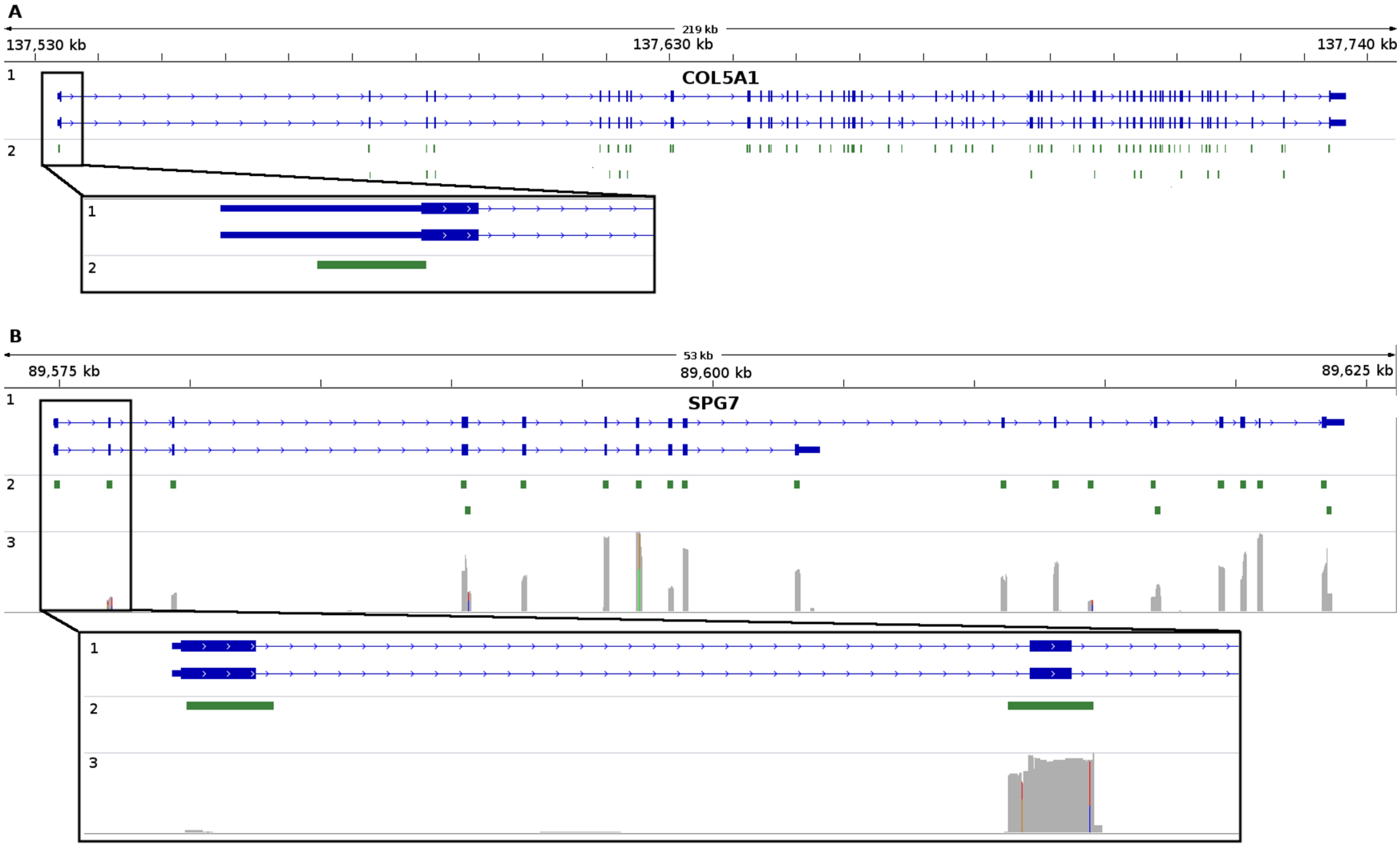

Supplementary figure 2

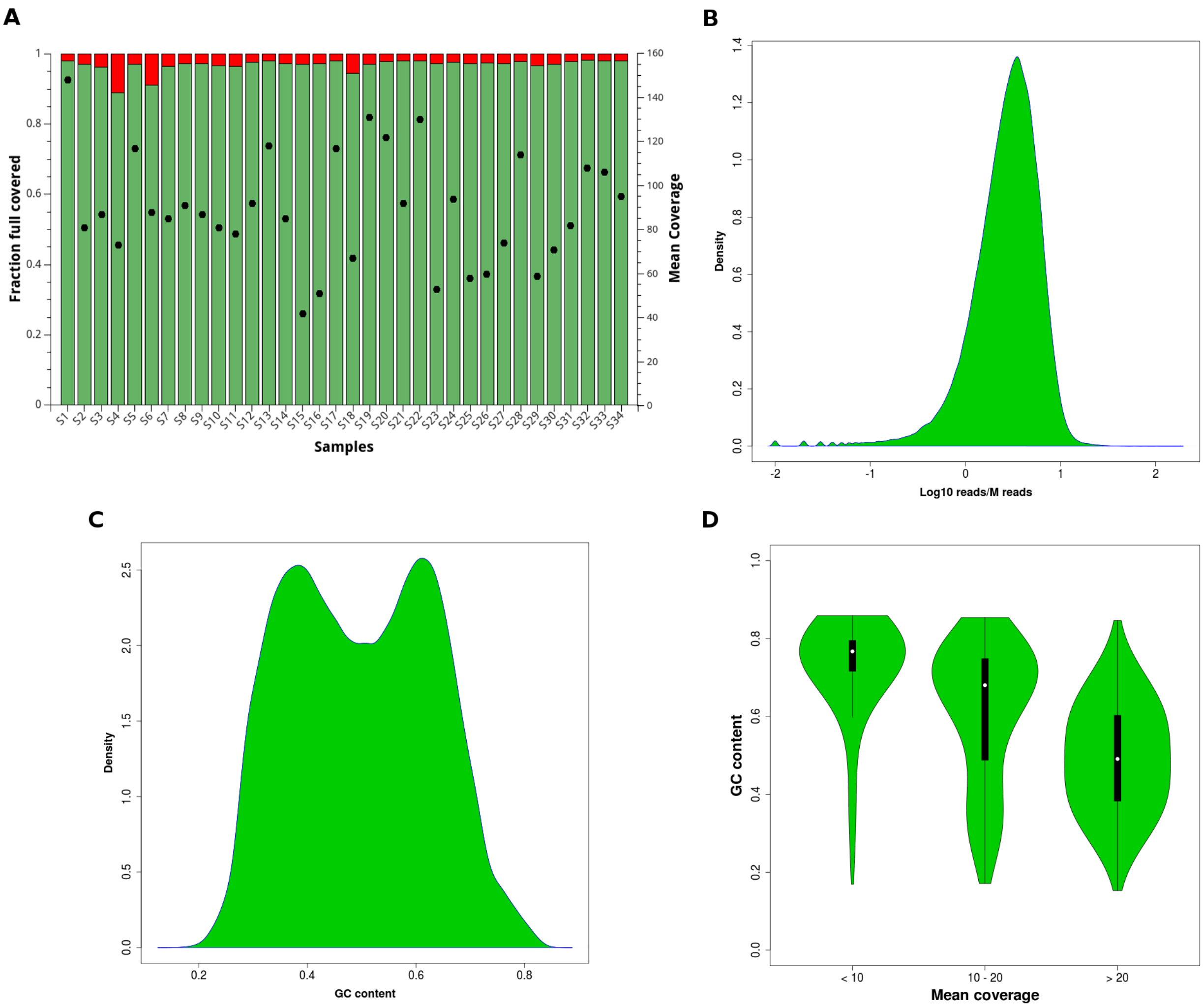

Supplementary figure 3

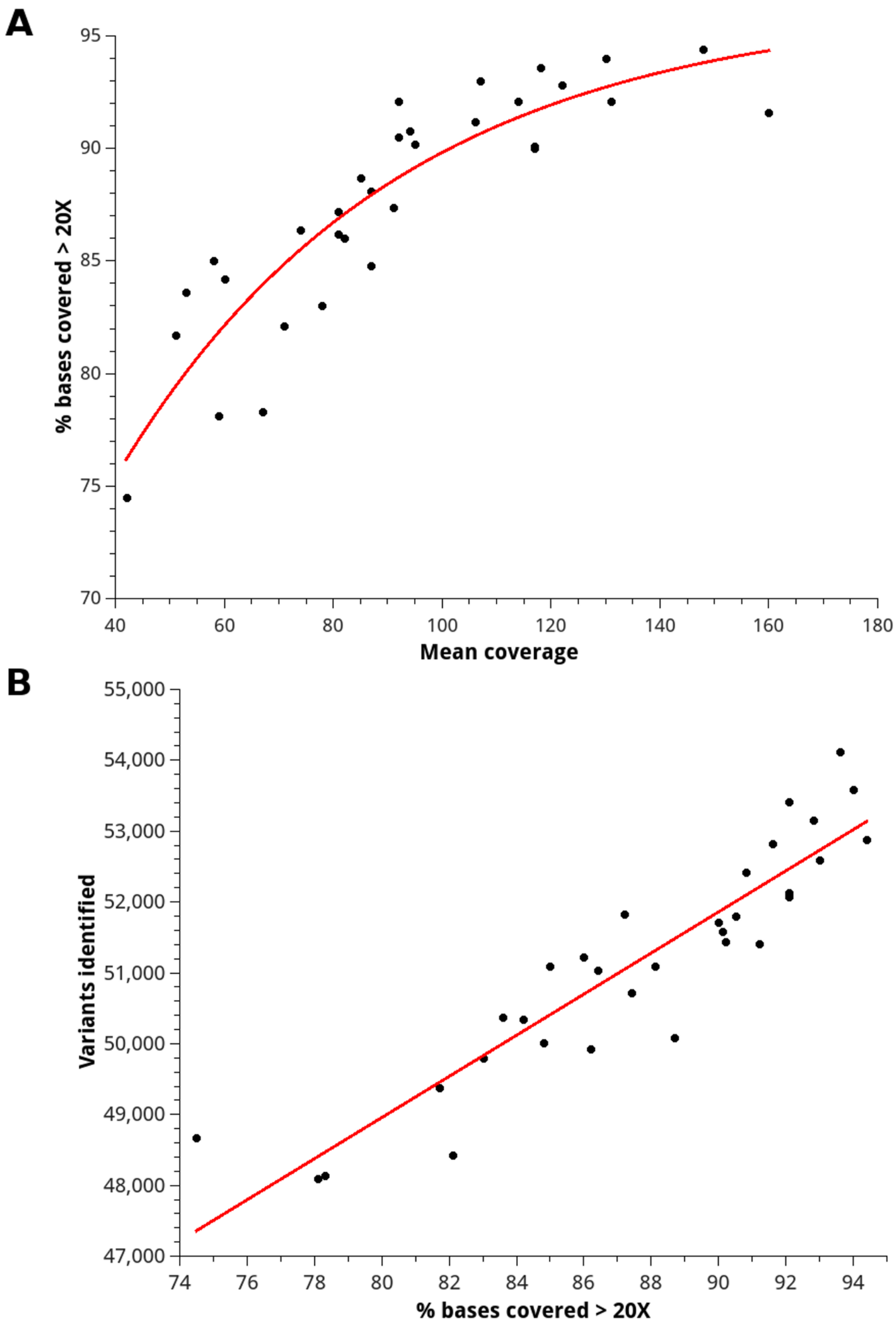

Supplementary figure 4

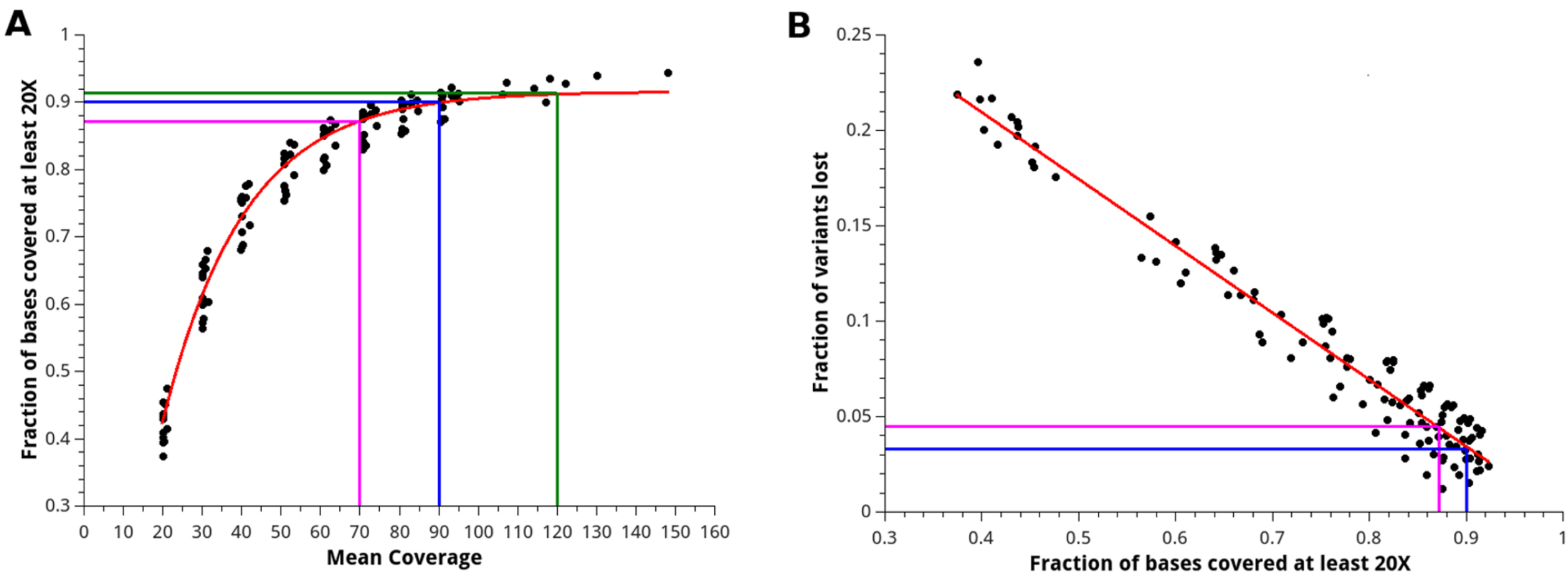

Supplementary figure 5

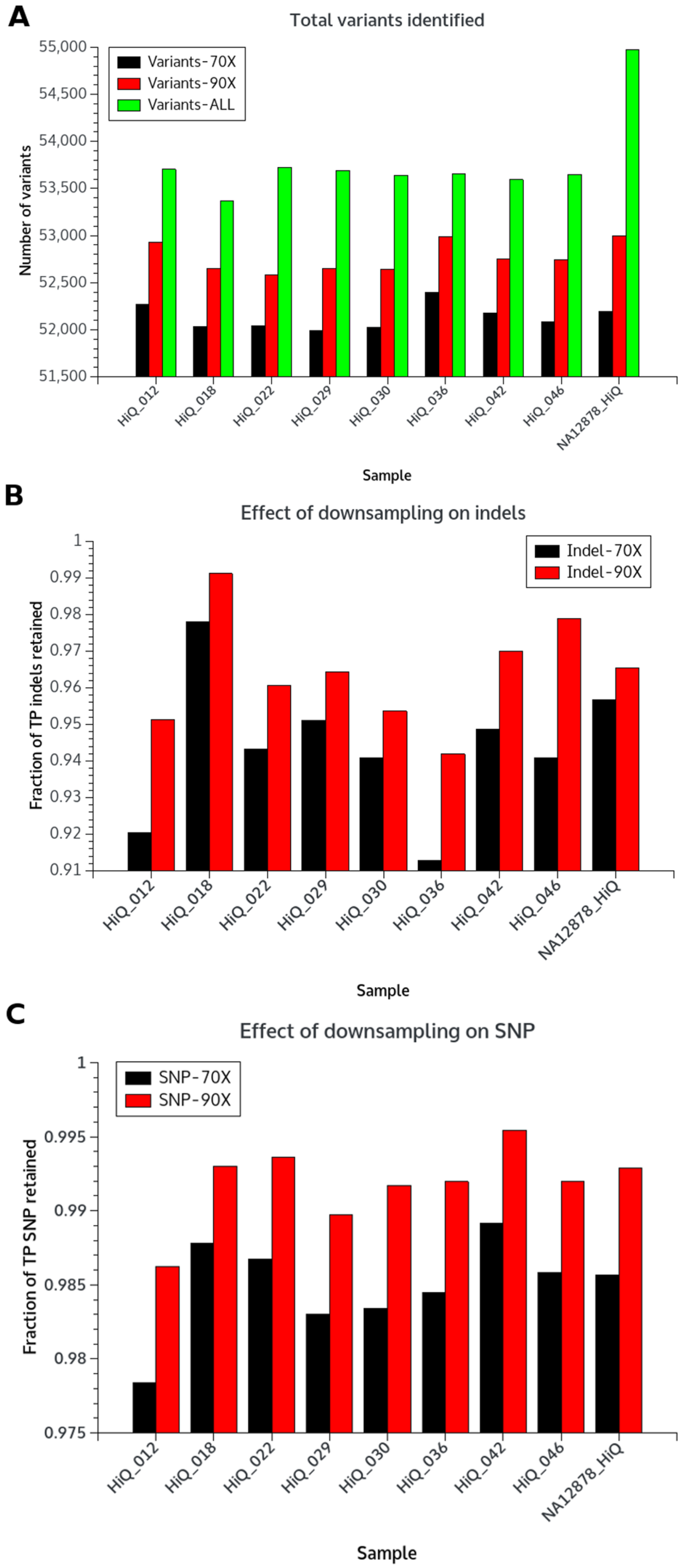

Supplementary figure 6

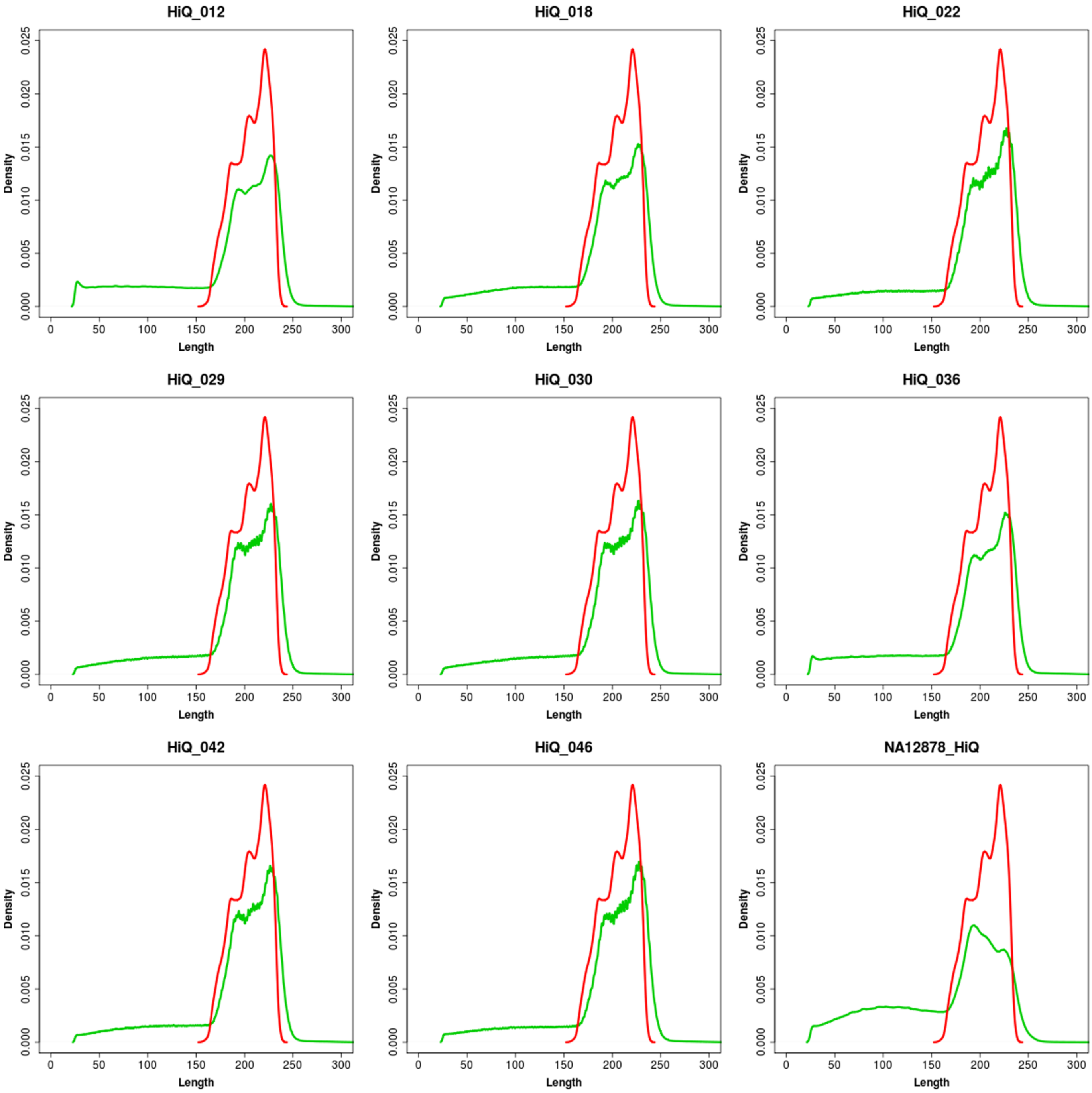

**Supplementary figure legends**

**Supplementary figure 1. Example of medical relevant genes not completely addressed**
We reported as example the COL5A1 gene on chromosome 9 (A)  and SPG7 gene on chromosome 16 (B), implicated in Enhlers-Danlos syndrome and Spastic Paraplegia, respectively. Genomic coordinates and dimension of the region are reported above the gene structure (track 1). Placement of the AmpliSeq Exome amplicons is reported track 2 and the read depth at each position in track 3. Expansion of the first exon in (A) shows a misplaced amplicon, while expansion in (B) shows that the first exon is addressed by the amplicon, but fail to be sequenced.

**Supplementary figure 2. Coverage analysis.**
(A) For each sample sequenced the mean coverage is reported with black dots while the fraction of human CDS bases covered and uncovered is represent in green and red, respectively. Analysis of coverage uniformity across all amplicons across the sequenced samples revealed good uniformity as suggested by the density plot of the fraction of reads captured by each amplicon (B). Most of the designed amplicons have a medium % GC content as shown in density plot of GC content (C). The content of GC has a strong influence on sequencing results and GC-rich amplicons tends to fail in sequencing, as showed by the violin plot in (D), analyzing the GC content of amplicons with low mean coverage across all the sequenced samples

**Supplementary figure 3. Sequencing results**
The Gb sequenced for each sample and the corresponding fraction of bases covered at least 20X are represented in (A), while the relationship between the number of variants identified and the read depth is represented in (B). In both panels the red line represents the trend line of data distribution.

**Supplementary figure 4. Analysis of coverage effect on variant calling**
The effect of coverage metrics on variant calling was studied by downsampling the analyzed samples. The fraction of bases covered at least 20X is exponentially related to the mean coverage (A) and reach a maximum above 120X. This parameter is also a predictor of the performance in variant identification since it is directly related to the fraction of variant missed (B). In both panels straight lines represent the coverage levels expected when sequencing 2 (green), 3 (blue) or 4 (magenta) samples on a single PI v3 chip in optimal conditions. Trends lines of data distributions are shown in red

**Supplementary figure 5. Downsampling on HiQ dataset**
We determined the number of variants identified in the 9 HiQ dataset from NA12878 (A) using the full set of sequencing data (green bars) or downsampled data at mean coverage of 90X (red bars) or 70X (black bars). We also calculated the fraction of true positive indels (B) and SNPs (C) retained when analyzing 90X (red bars) and 70X (black bars) downsampled dataset.

**Supplementary figure 6. Read length profiles of HiQ datasets**
The read length profiles from each of the 9 HiQ datasets is reported as density plots. The read length profile of each sample is represented by green line, while the distribution of amplicon lengths expected from the BED file of AmpliSeq Exome kit is reported in red. Significant loss of long fragments could be observed in NA12878_HiQ dataset.
